# Current and projected future economic burden of Parkinson’s disease in the U.S.

**DOI:** 10.1038/s41531-020-0117-1

**Published:** 2020-07-09

**Authors:** Wenya Yang, Jamie L. Hamilton, Catherine Kopil, James C. Beck, Caroline M. Tanner, Roger L. Albin, E. Ray Dorsey, Nabila Dahodwala, Inna Cintina, Paul Hogan, Ted Thompson

**Affiliations:** 1 The Lewin Group, Falls Church, VA 22042 USA; 2 The Michael J. Fox Foundation, New York, NY 10120 USA; 3 Parkinson’s Foundation, New York, NY 10018 USA; 4 University of California, San Francisco, CA 94115 USA; 5 University of Michigan, Ann Arbor, MI 48109 USA; 6 VA Ann Arbor Health System, Ann Arbor, MI 48105 USA; 7 University of Rochester, Rochester, NY 14642 USA; 8 Penn Parkinson’s Disease and Movement’s Disorders Center, Philadelphia, PA 19107 USA

**Keywords:** Health care economics, Neurological disorders

## Abstract

Parkinson’s disease (PD) is one of the world’s fastest growing neurological disorders. Much is unknown about PD-associated economic burdens in the United States (U.S.) and other high-income nations. This study provides a comprehensive analysis of the economic burdens of PD in the U.S. (2017) and projections for the next two decades. Multiple data sources were used to estimate the costs of PD, including public and private administrative claims data, Medicare Current Beneficiary Survey, Medical Expenditure Panel Survey, and a primary survey (*n* = 4,548) designed for this study. We estimated a U.S. prevalence of approximately one million individuals with diagnosed Parkinson’s disease in 2017 and a total economic burden of $51.9 billion. The total burden of PD includes direct medical costs of $25.4 billion and $26.5 billion in indirect and non-medical costs, including an indirect cost of $14.2 billion (PWP and caregiver burden combined), non-medical costs of $7.5 billion, and $4.8 billion due to disability income received by PWPs. The Medicare program bears the largest share of excess medical costs, as most PD patients are over age 65. Projected PD prevalence will be more than 1.6 million with projected total economic burden surpassing $79 billion by 2037. The economic burden of PD was previously underestimated. Our findings underscore the substantial burden of PD to society, payers, patients, and caregivers. Interventions to reduce PD incidence, delay disease progression, and alleviate symptom burden may reduce the future economic burden of PD.

## Introduction

Parkinson’s disease (PD) is a slowly progressive neurodegenerative disorder affecting approximately one million Americans^1^. In addition to debilitating features of PD itself such as tremor, bradykinesia, anxiety, depression, and cognitive impairments, persons with PD (PWPs) experience significant comorbidities, including increased rates of infections, cardiac and gastrointestinal disorders, and fall-related injuries^2–5^. PWPs have higher medical care needs, lose the ability to work, often miss work, and require the assistance of paid and unpaid care partners. These indirect effects create ripple economic burdens. Whetten-Goldstein, for example, reported that family care partners, particularly spouses, spend an average of 22 h per week providing care to PWPs. The direct and indirect economic burdens of PD are likely to be substantial^6^.

Although PD is one of the world’s fastest growing neurological disorders^7^, much is unknown about its current economic burden in the United States (U.S.) and other high-income nations. The objective of this study is to estimate the economic burden of PD in the U.S. in 2017 and to project its probable economic impact over the next 20 years (from 2018 to 2037). We used a human capital approach, assigning monetary values to loss of health as the lost value of economic productivity due to illness, disability, or premature mortality. We aim to provide the most comprehensive estimate of the total economic burden of PD from a societal perspective and reduce knowledge gaps in less well-understood cost components, including future earnings losses due to premature death, productivity losses both in the labor market and in social life, and care partner burdens.

As there is no one dataset that addresses all the costs associated with PD, we relied on several data sources to conduct these analyses. These include large, comprehensive, and nationally representative claims databases from public and private payers, and a primary survey—the Social and Financial Impact of Parkinson’s Disease Survey (hereafter, PD Impact Survey)—to estimate indirect productivity losses for PWPs and care partners, disability benefits, and non-medical costs associated with PD, such as home renovations, motor vehicle modifications, and expenditures for daily non-medical care. See the “Method” section for more details.

## Results

We estimated that 1.04 million individuals in the U.S. have been diagnosed with PD in 2017, with about 919,000 (89%) eligible for Medicare (91% age ≥65; 9% <65). PD was more prevalent in the ≥65 population (16.9 cases vs. 0.7 per 1,000 population), and more prevalent in men (595,000 males vs. 443,000 females). See Supplementary Table 1 for details.

**Fig. 1:**
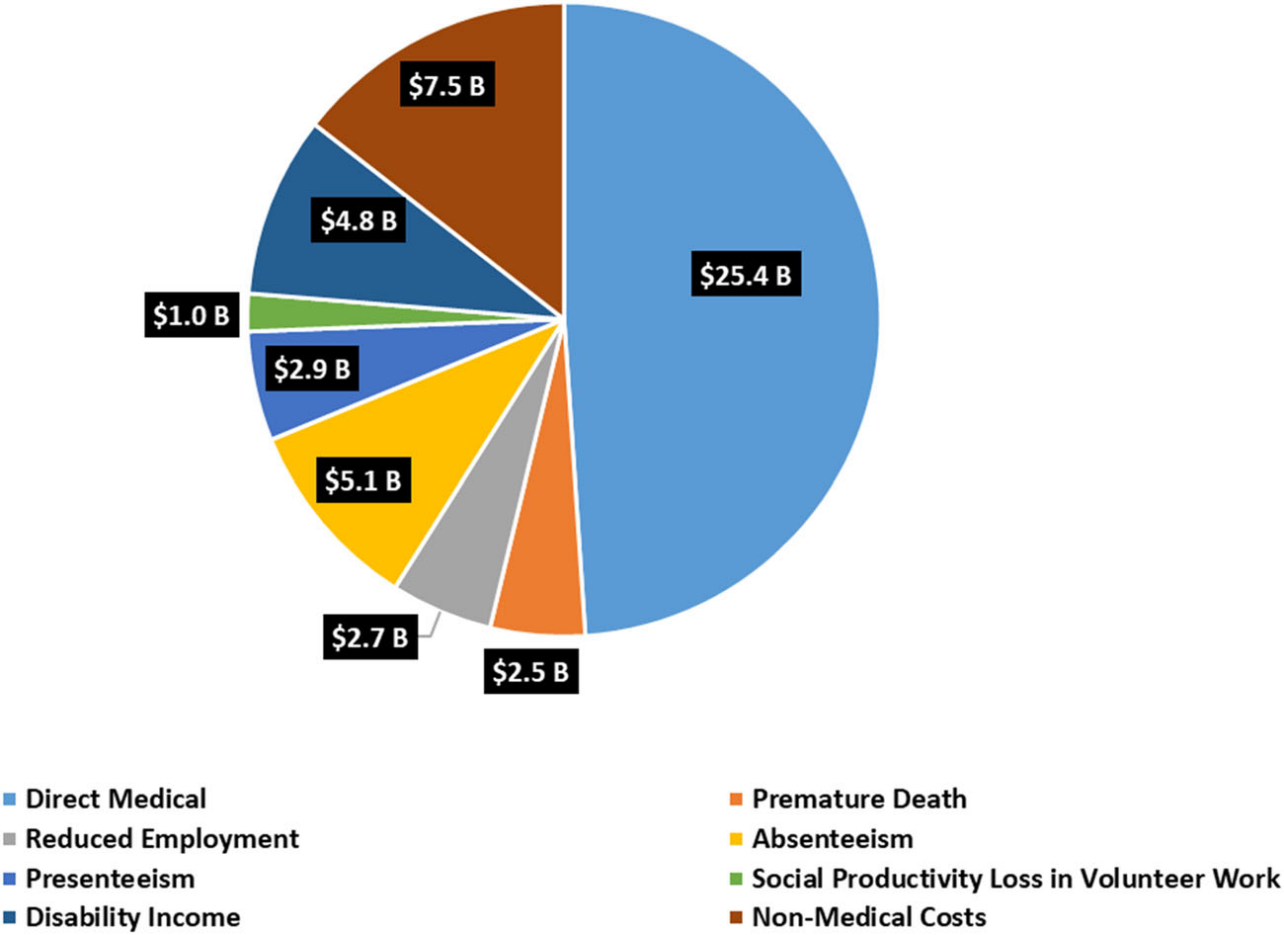
Total economic burden of Parkinson’s disease in the U.S. in 2017 (in billions). Pie chart of the economic burden of Parkinson’s disease in the U.S. in 2017 by components (in billion $s).

PD is associated with substantial excess costs in direct medical costs, indirect productivity losses, non-medical costs, and disability income. Figure 1 shows the estimated total economic burden of PD in the U.S. in 2017 by cost components. The total economic burden of PD was $51.9 billion, including direct medical costs of $25.4 billion and $26.5 billion in indirect and non-medical costs. The latter included total indirect costs of $14.2 billion (including future earnings losses due to premature death, reduced employment, absenteeism [disease-related number of days missed from work], presenteeism [disease-related unproductive work days while working], and social productivity losses in volunteer work), non-medical costs of $7.5 billion (including paid daily non-medical care, home modifications, motor vehicle modifications, and other expenses), and $4.8 billion due to disability income received by PWPs.

The excess direct medical cost per PWP attributed to PD was calculated as the difference between the total annual average medical cost of PWPs and that of matched control groups (based on age, gender, race/ethnicity, and insurance). As shown in Table 1, and consistent with the higher prevalence estimate among the older population, 90% of the direct cost of $25.4 billion in 2017 was borne by populations eligible for Medicare coverage, 7% by those with private insurance, and 3% by other coverage (i.e., PWPs with other insurance, such as Medicaid or Department of Veterans Affairs [VA] coverage, or without insurance coverage). Small sample size prevented further breakdown of this last group. Hospital inpatient care, non-acute institutional care (including skilled nursing facility [SNF], nursing home, hospice), and outpatient and ancillary care represented the three largest cost categories. The average direct cost per PWP was higher for PWPs eligible for Medicare (all ages) than those age <65 and not covered by Medicare.

**Table 1:** Direct medical cost of Parkinson’s disease by age, gender, insurance coverage, and types of service (in 2017 $s).

	Total excess medical cost due to PD		Per PWP ($)
	(in Million $s)	Percentage of the total	
Age			
≤49	490	2%	29,346
50–64	4,153	16%	22,598
65–74	8,858	35%	23,011
≥75	11,847	47%	26,222
Gender			
Male	13,580	54%	22,838
Female	11,768	46%	26,589
Insurance			
Private	1,742	7%	22,671
Medicare	22,793	90%	24,811
Other^a^	812	3%	19,489
Type of service			
Non-acute institutional care	7,144	28.2%	6,888
Hospital inpatient	7,190	28.4%	6,932
Outpatient	5,506	21.7%	5,308
Physician office	1,226	4.8%	1,182
Durable medical equipment	145	0.6%	140
Prescription medication	4,137	16.3%	3,988
Overall	25,348	100%	24,439

Source: Authors’ estimation of PD prevalence using 2011–2015 Medical Expenditure Panel Survey (MEPS), 2015 Medicare Current Beneficiary Survey (MCBS), and Census population projections for 2017; combined with direct medical cost estimated using 2016 Optum claims data, 2015 Medicare Standard Analytical File 5% sample claims, and 2015 MCBS.

^a^Other includes Medicaid, other insurance, and uninsured. Sample size in MEPS did not support further breakdown.

Table 2 shows the estimated indirect and non-medical costs of PD in more detail. Among the total indirect and non-medical costs of $26.5 billion, nearly $20 billion was attributed to PWPs and more than $6.5 billion was due to PD-related productivity losses among unpaid care partners. For PWPs, the three largest indirect cost categories were future earnings loss due to PD-related premature death, earnings loss from reduced employment, and absenteeism. Paid daily non-medical care and expenses related to home modification—represented the largest non-medical costs. Other disability income was the largest share of the disability income cost component. For the care partners, on-the-job productivity loss due to absenteeism and presentism surpassed that of PWPs because care partners on average were younger and more likely to be employed (13% employed among PWPs vs. 28% employed among primary and 66% among secondary, care partners). Per PWP indirect and non-medical costs (PWP and care partner costs combined) were slightly higher than the per PWP direct medical cost ($25,558 vs. $24,439).

**Table 2:** The indirect and non-medical cost of Parkinson’s disease by cost component (in 2017 $s).

	Total indirect and medical costs (in million $s)			Per PWP ($)		
	PWP loss	Care partner loss	PWP & care partner	PWP loss	Care partner loss	PWP & care partner
Premature death	2,508	NA	2,508	2,418	NA	2,418
Reduced employment	1,873	802	2,675	1,806	773	2,579
Absenteeism	1,395	3,655	5,050	1,345	3,524	4,869
Presenteeism	1,263	1,684	2,946	1,217	1,623	2,841
Social productivity loss in volunteer work	623	410	1,034	601	396	997
Disability income						
Supplemental security income (SSI)	561	NA	561	541	NA	541
Social security disability insurance (SSDI)	1,677	NA	1,677	1,617	NA	1,617
Other disability income^a^	2,521	NA	2,521	2,431	NA	2,431
Non-medical costs						
Paid daily non-medical care	3,847	NA	3,847	3,709	NA	3,709
Home modification	2,232	NA	2,232	2,151	NA	2,151
Motor vehicle modification	931	NA	931	897	NA	897
Other expenses	527	NA	527	508	NA	508
Overall	19,958	6,551	26,509	19,242	6,316	25,558

Source: Authors’ analyses of the PD Impact Survey data, supplemented with other data sources such as Centers for Disease Control and Prevention Wide-ranging Online Data for Epidemiologic Research (CDC WONDER) death records, Bureau of Labor Statistics (BLS) earnings data; combined with prevalence estimated using 2011–2015 Medical Expenditure Panel Survey (MEPS), 2015 Medicare Current Beneficiary Survey (MCBS), and Census population projections for 2017.

^a^Includes other disability income sources such as VA disability compensation, government employee disability compensation, and state disability insurance or personal disability insurance payments.

**Fig. 2:**
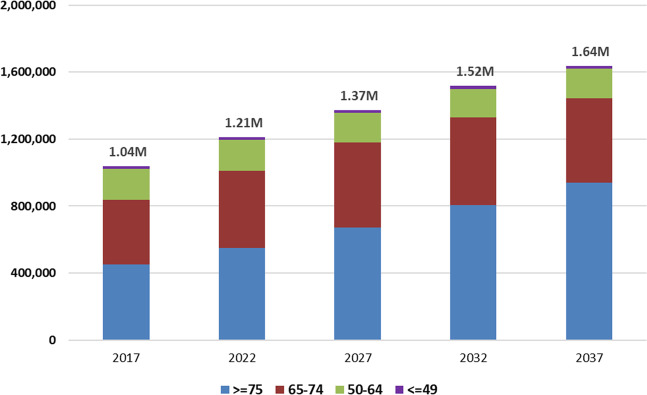
Projected number of persons with diagnosed Parkinson’s disease in the U.S. by 2037. Bar chart of the projected number of persons with Parkinson’s disease in the U.S. by 2037. Authors’ projections by applying PD prevalence estimated for 2017, using 2011–2015 Medical Expenditure Panel Survey (MEPS), 2015 Medicare Current Beneficiary Survey (MCBS), and Census population projections for 2017, to future U.S. population projections from the year 2018–2037. Projections for other years between 2018 and 2037 are available upon request.

We projected the future prevalence of PD by applying estimated age- and gender-specific PD prevalence rates in 2017 to U.S. Census Bureau population projections for years 2018–2037. According to the Census Bureau population projection, from year 2017 to 2037, the U.S. total population will increase by about 14% (from ~326 million to ~372 million) while the ≥65 population will increase by about 62% (from ~50 million to ~80 million). Due to the much higher PD prevalence rate among the older U.S. population, PD prevalence will increase significantly over the next two decades. As shown in Fig. 2, the number of PWPs will increase from 1.04 million in 2017 to about 1.09 million in 2018, and to about 1.64 million in 2037. The number of PWPs will increase disproportionately in the 75 and older age group.

**Fig. 3:**
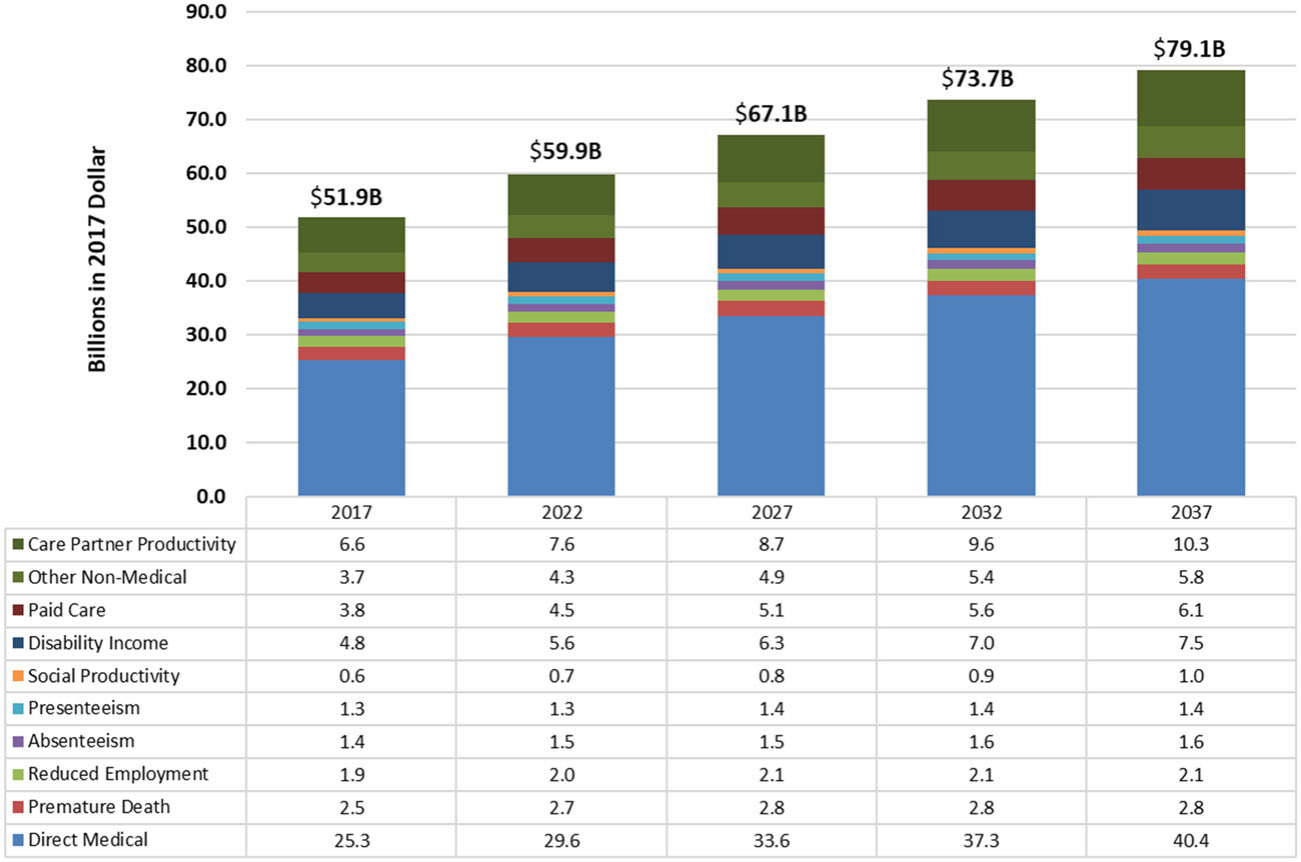
Projected economic burden of Parkinson’s disease in the U.S. by 2037 (billions in 2017 $). Bar chart of the projected economic burden of Parkinson’s disease in the U.S. by 2037. Authors’ projections by applying estimated 2017 direct medical cost using 2016 Optum claims data, 2015 Medicare Standard Analytical File 5% sample claims, and 2015 MCBS, and the indirect and non-medical costs estimated using data sources such as the CDC WONDER files, BLS earnings data, and responses from the PD Impact Survey, to projected future PD prevalence. Future PD prevalence was estimated by applying PD prevalence in 2017, estimated using 2011–2015 Medical Expenditure Panel Survey (MEPS), 2015 Medicare Current Beneficiary Survey (MCBS), and Census population projections for 2017, to future U.S. population projections from year 2018 to 2037.

The future overall economic impact of PD through the year 2037 was projected by combining the projected future number of PWPs with the estimated 2017 cost burden component per PWP. As shown in Fig. 3, the total economic burden of PD would increase from $51.9 billion in 2017 to be about $54.2 billion in 2018 and about $79.1 billion in 2037. Among all cost components, total direct medical costs and social productivity loss would increase by about 52% from 2017 to 2037. Disability income, costs for paid care, other non-medical costs, and care partner productivity loss would increase by about 50%. Costs secondary to premature death, reduced employment, absenteeism, and presenteeism would increase by 9%-15%. These projections represent the future economic burden in real 2017 dollars.

## Discussion

This study provides a comprehensive assessment of the economic burdens of PD in the U.S. We updated cost components included in previous studies, and captured cost components omitted from previous research.

We estimated a total of 1.04 million individuals with diagnosed PD in the U.S. in 2017 with an associated $51.9 billion in total economic costs. We estimated direct medical costs of $25.4 billion and indirect and non-medical costs of $26.5 billion. We found that more than 90% of the direct cost was borne by populations eligible for Medicare coverage. Across different types of health services, hospital inpatient care (28.4%), non-acute institutional care (28.2%), and outpatient care (21.7%) represent the three largest shares of the direct medical cost. The total indirect and non-medical costs of $26.5 billion consisted of $7.7 billion in PWP productivity loss, $7.5 billion of non-medical cost, $4.8 billion in disability income, and $6.6 billion in unpaid care partners’ productivity loss. Projected PD prevalence will be more than 1.6 million with total economic burden surpassing $79 billion by 2037. Our estimate of a 58% increase from 1.04 million to 1.6 million in the total number of PWPs from 2017 to 2037 is slightly lower than, but comparable to, several prior U.S.-based or international projections^1,8–10^.

PD increases economic burden more than previously understood and affects PWPs, care partners, employers, payers, and society at large. Our estimated prevalence, annual direct medical costs, and indirect and non-medical costs are significantly higher than several previous U.S. based studies assessing the economic burden of PD (Table 3)^8,11,12^. Our prevalence estimates are comparable to the more recent estimates of Marras et al.^1^. Our direct medical cost estimate is very similar to the recent estimate of Mantri et al.^13^. They estimated that Medicare spends an average $20,142 (in 2014) per year for PWP beneficiaries with Medicare Part A & B coverage. Comparisons between our study and these prior estimates indicate that differences in prevalence and cost estimates result mainly from several methodological differences.

**Table 3:** Comparison of the current study with previous U.S. studies estimating the burden of Parkinson’s disease.

U.S. PD burden study	Prevalence	Total cost	Per capita cost	Study design	Key data sources for prevalence, direct costs, & indirect/non-medical costs	Cost components
Current study	1,040,000 (in 2017)	Direct: $25,348 M Indirect & non-medical: $26,509 M Overall: $51.8 B in 2017	Direct: $24,439 (in 2017) Indirect & non-medical: $25,558 (in 2017)	Direct costs: calculated as the cost difference between a PD sample and a comparison group matched based on age, gender, race/ethnicity, and insurance; Indirect & non-medical costs: calculated using a PD-specific survey	Prevalence: 2011–2015 MEPS, 2015 MCBS Direct costs: claims data for both privately insured and Medicare populations Indirect & non-medical costs: comprehensive primary survey (The PD Impact Survey)	Medical & prescription drug costs, premature death, unemployment, absenteeism, presentism, social productivity loss, disability payments, informal & formal care, and non-medical expenses
Kowal et al.^8^	630,000 (in 2010)	Direct: $8,064 M Indirect & non-medical: $6,327 M Overall: $14.4 B in 2010	Direct: $12,805 in 2010 ($15,749 in 2017) Indirect & non-medical: $10,046 in 2010 ($12,355 in 2017)	Direct costs: estimated using national surveys and a cost-allocation approach. Indirect & non-medical costs: calculated mainly using NHIS with regressions	Prevalence: 2003–2008 MEPS, 2004 NNHS Direct costs: service-specific national survey data such as NAMCS or NHAMCS Indirect & non-medical costs: 2008 NHIS; the 2010 MetLife Survey of Adult Day Services	Medical & prescription drug costs, reduced employment, absenteeism, reduced household income, disability payments, adult day care, and misc household expenditure
O’Brien et al.^11^	About 500,000 (in 2007)	Direct: $6,246 M Indirect & non-medical: $4,568 M Overall: $10.8 B in 2007	Direct: $12,491 in 2007 ($15,823 in 2017) Indirect & non-medical: $9,135 in 2007 ($11,572 in 2017)	Direct costs: estimated using health encounter data and a cost modeling approach. Indirect & non-medical costs: estimated using national surveys and published literature	Prevalence: Assumption made based on range provided in literature. Direct costs: healthcare encounter data from 6 states Indirect & non-medical costs: NHIS-D 1995, HHCS 2006–2007, Parrish et al.^45^, Whetten-Goldstein et al.^6^	Medical & prescription drug costs, absenteeism (patient and caregiver), out-of-pocket expenses for in-home personal care, and Social Security death benefit
Huse et al.^12^	645,000 (in 2002)	Direct costs: $6,675 M Indirect & non-medical costs: $16,335 M Overall: $23.0 B in 2002	Direct: $10,349 in 2002 ($16,123 in 2017) Indirect & non-medical: $25,326 in 2002 ($39,457 in 2017)	Direct costs: estimated using regression adjusting for demographics and comorbidities. Indirect & non-medical costs: extrapolated from previous literature based on a PD-specific survey	Prevalence: Extrapolation from the EUROPARKINSON study Direct costs: Medstat Marketscan commercial and Medicaid claims data 1999–2002 Indirect & non-medical costs: Whetten-Goldstein et al.^6^ (the Duke Survey for PD *N* = 109)	Medical & prescription drug costs, productivity losses, informal and formal care, non-medical expenses

Source: Authors’ analysis as compared with previous literature.

*NAMCS* National Ambulatory Medical Care Survey, *NHAMCS* National Hospital Ambulatory Medical Care Survey, *HHCS* Hospital and Healthcare Compensation Services, *NHIS* National Health Interview Survey, *NHIS-D* National Health Interview Survey on Disability, *NNHS* National Nursing Home Survey, *MEPS* Medical Expenditure Panel Survey, *OOP* out-of-pocket.

Our study used the more recent data for prevalence (i.e., 2011–2015 Medical Expenditure Panel Survey (MEPS) data combined with the 2015 Medicare Current Beneficiary Survey (MCBS)), and is likely more representative of the Medicare beneficiary population because MCBS is specifically designed for Medicare beneficiaries. Kowal et al. estimated U.S. PD prevalence of 647,000 in 2010 from 2003 to 2008 MEPS data combined with the 2004 National Nursing Home Survey (NNHS) for the institutionalized population^8^. O’Brien et al. assumed that U.S. PD prevalence was roughly 500,000 in 2007^11^. Huse et al. applied estimated PD prevalence rates from a European study to the U.S. population^12^.

Differences in estimation methods likely contributed to differences in cost estimates. We used claims data and calculated PD attributable direct costs as the cost difference between a PD sample and a comparison group matched based on age, gender, race/ethnicity, and insurance. Kowal et al. used a cost-allocation approach and data from national surveys that excluded some direct medical costs such as outpatient lab tests or durable medical equipmen^8^. The Huse et al. analysis is the most comparable to our study as they also mainly used claims data for direct costs (1999–2002 MarketScan claims database) and a regression-based approach to estimate the cost increase due to PD^12^. O’Brien used a cost modeling approach to estimate the direct medical cost of PD using healthcare encounter data (e.g., inpatient admission, emergency room visit) representing limited geographic areas^11^. The Noyes et al. study was conducted a decade prior to our study and adjusted for demographic factors and comorbidities (e.g., cardiovascular, psychiatric, stroke, etc.)^14^. Our study adjusted for age, gender, race/ethnicity, and insurance coverage based on the understanding that PD affects the whole body and increases healthcare utilization and spending for comorbidities. Similarly, Dieleman et al. estimated a much smaller total direct medical cost of PD $5.5 billion in 2017 dollars, but PD-related comorbidity costs were allocated to those comorbidities, not PD^15^.

While most prior studies largely relied on measures available from secondary data sources to estimate indirect and non-medical costs, our study relied on a comprehensive primary survey—the PD Impact Survey—specifically designed for a comprehensive analysis of PD costs. This is the largest survey about the economic burden of PD with a comprehensive set of cost components not routinely available from secondary sources. Although patient self-reported survey outcomes are subject to potential selection bias or recall bias, the large sample size likely mitigates some of these issues. Other studies included fewer cost components that were available in secondary data sources such as national surveys^8,12^ or relied on smaller PD-specific surveys^6,11^.

Our annual total cost estimate per PWP is similar to cost estimates for other chronic, disabling neurodegenerative diseases in the U.S. The annual per person cost of dementia, including cost of medical care and cost of informal care was estimated to be $69,230 in 2017 dollars^16^. Another study found that the annual per person cost (including direct medical, non-medical, and indirect costs) for amyotrophic lateral sclerosis was $78,334 in 2017^17^.

A limitation of the current study is the omission of undiagnosed PD (about 12 to 42% of all PWPs), which may lead to costs understatement^18–21^. A second potential limitation is related to how we identified PD cases for the prevalence estimate. We relied on any one diagnosis of PD in the MEPS data to identify non-Medicare <65 PD cases. This may have included some false positives and left out some false negatives. The impact of potential misclassification would be small considering that ~10% of the PD cases were <65 years and non-Medicare eligible. Nearly 90% of the 1.04 million (weighted) PWPs eligible for Medicare were identified via patient self-report of PD in the MCBS data. Patient self-reporting of PD diagnosis was shown to have excellent accuracy when compared with clinician diagnosis^22^. We conducted a sensitivity analysis by using only one occurrence of PD diagnosis to identify PD cases in claims data, which led to a slightly lower total direct cost estimate ($25 billion) relative to the more stringent criteria requiring ≥1 diagnosis in inpatient setting and ≥2 in non-inpatient setting ($25.4 billion). The publicly available MEPS files only contain 3-digit ICD-9 diagnosis codes and the ICD-9 code 332 may contain 10–15% PD mimics^23^. The impact of including PD mimics is hard to assess as several forms of PD mimics are more severe than PD and would involve more healthcare encounters (e.g., physical therapy). However, these patients may incur lower medication costs as there are no specific medications.

A third limitation is the small sample of PWPs identified from 5-year combined MEPS data (*n* = 245 with 55 PWPs age <65) and from the 2015 MCBS (*n* = 229). This is a potential source of poor precision and biases inherent in small samples. Analysis of racial and ethnic differences in cost burdens would help understanding the disparities of PD burden, but the small sample sizes for groups other than Non-Hispanic White prevented breakdowns of costs by race/ethnicity. Future studies using more comprehensive approaches may provide more precise insights, or allow investigation by geographic region or demographic subgroup.

Another limitation is that, due to data limitations, we used private insurance claims to impute costs for the ~42,000 PWPs (4% of the 1.04 million PWPs in 2017) in the Other group (non-private, non-Medicare PWPs age <65). To ensure that any intrinsic cost difference between the privately insured and the non-private, non-Medicare population are accounted for, we used the MEPS data and a regression analysis (see the “Methods”) to estimate the cost ratio between the two groups (regardless of their PD status) and used the cost ratio to adjust the imputed costs. These imputed costs may be misleading to the extent that this subgroup is receiving different level and types of medical services than the privately insured.

Finally, we may have underestimated the long-term care cost because of lack of Medicaid-specific data. As nearly 90% of the PWPs identified were eligible for Medicare, the potential under- or over-estimation of long-term care costs would only affect the ~42,000 PWPs in the Other group, to the extent that their long-term care costs are different from that of the privately insured. For the nearly 90% of Medicare beneficiaries, we used the MCBS to estimate long-term care costs. The MCBS captures health services use and costs incurred by Medicare beneficiaries, whether or not Medicare is the primary payer. A 2015 study^24^ found that Medicare alone had ~100,000 institutionalized PWPs. The average annual costs of nursing home care was between $82,128 and $92,376 per-person-per-year in 2019 (https://www.seniorliving.org/nursing-homes/costs/). Taken together, the annual nursing home care cost would be $8–$10 billion for the PWPs eligible for Medicare. This is somewhat higher than our estimate (Table 1), but this range is the total cost of nursing home care spent for Medicare PWPs, not necessarily the excess cost due to PD for long-term care, because some PWPs would incur long-term care costs for other reasons.

Our projections, applying estimated 2017 PD prevalence, are cautious. Some factors, such as declining tobacco abuse and rising longevity, could lead to increases in PD incidence and prevalence^7,25^. Effective public health and/or healthcare intervention strategies could reduce the prevalence and mitigate the economic burdens of PD. Preventive measures reducing environmental factors linked to PD risk might reduce PD incidence^26–32^. Therapies that prevent or delay the progression of PD are the most important unmet medical need in PD therapeutics. Symptomatic therapies focusing on treatment of PD features such as falls and cognitive impairments might also significantly reduce the economic burdens of PD. Finally, increasing access to specialist care might also reduce the PD burden as there is evidence that specialist care improves patient clinical outcomes and reduces healthcare utilization, costs, and mortality^33^. Health improvement gained from such interventions may significantly reduce the future economic burden of PD.

Our results underscore the need for preventive or treatment measures that directly reduce the prevalence and/or impacts of PD, and for policy initiatives to better support affected individuals and families, improve disease management, provide work-site support, and enhance employment and occupational training. The study findings could help inform decision making in PD-related health resource investments and research prioritization.

## Methods

**Fig. 4:**
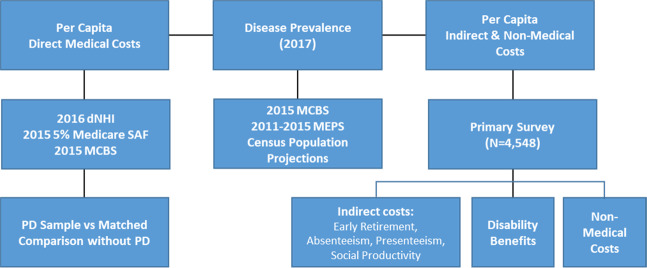
Flow chart of cost calculation and data sources. Flow chart showing data source and cost calculation steps. Abbreviations: dNHI: Optum de-identified Normative Health Information system; Medicare SAF: Medicare Standard Analytical File; MCBS: Medicare Current Beneficiary Survey; MEPS: Medical Expenditure Panel Survey.

We used a prevalence-based approach in estimating the economic burdens of PD in 2017. The estimated number of PWPs in 2017 was combined with per-capita cost to derive national economic burden by population characteristics. Figure 4 shows the cost calculation steps and data source for each cost component.

### PD prevalence

Using Census Bureau population projections combined with the MCBS and the MEPS data, we calculated the age-gender-insurance specific prevalence rates and applied them to the 2017 U.S. population. Due to the very small sample size, the population over age 65 who are covered by non-Medicare insurance were assigned to the Medicare group.

The MCBS is a continuous survey of a representative national sample of 16,000 Medicare beneficiaries^34^. We used data from a single year, 2015, to estimate PD prevalence as the 2014 survey was not published. We identified the PWP sample from the MCBS based on survey questions such as “Since (month/year) has a doctor ever told you that you had Parkinson’s disease” or other condition screening questions contained in the chronic conditions dataset, the facility assessment dataset, or the Minimum Dataset (MDS). The identified PWPs were de-duplicated to obtain the unique number of individuals (*n* = 229) and weighted to obtain the total number of PWPs covered by Medicare.

For populations with other types of coverage, including the privately insured and Other (i.e., anyone covered by all other health plans such as Medicaid and VA, as well as those uninsured), we combined the 2011–2015 MEPS to increase the sample size. The MEPS does not have a survey question that could be used to identify PD. Therefore, we relied on the presence of any PD diagnosis in any of the MEPS chronic condition files to identify PWPs. The 3-digit ICD-9 diagnosis code included in the publicly available MEPS was used to identify PD (ICD-9 code 332), which may include about 10–15% of patients with secondary parkinsonism (ICD-9 332.1)^23^.

### Direct costs

To quantify the overall excess healthcare use due to PD, we compared the average healthcare costs of PWPs with that of a matched comparison group with similar characteristics but without PD and took the inter-group difference as the excess cost due to PD. For each PWP, 10 comparison individuals were matched based on age, gender, race/ethnicity, and insurance type. See Supplementary Table 2 for a comparison of patient characteristics.

Medical cost includes the amount paid by plan, patient, and third-party and was estimated by patient characteristics and types of healthcare services, including hospital inpatient stay, physician office visit, prescription medications, durable medical equipment, outpatient services (e.g., hospital outpatient care, physical therapy, occupational therapy, and all other ancillary services), and non-acute institutional care (including SNF, nursing home, hospice, and other similar services).

To estimate the direct medical cost of PD, we used three key data sources. For the privately insured population (<65 years of age), we used longitudinally-linked claims from the proprietary Optum de-identified Normative Health Information (dNHI) system. To ensure the completeness of the final claims, we used 2016 data (with a total membership of more than 30 million privately insured individuals) and required continuous full-year plan coverage to ensure completeness of health records.

For the Medicare-eligible population (including those age 65 and older and those <65 who were eligible for Medicare due to disability), we used the Medicare Standard Analytical File 5% sample claims data in year 2015 (the latest available at the time of this analysis). The Medicare 5% data includes both institutional and non-institutional claim types. The limitation of the Medicare 5% data is that it does not include Part D prescription drug claims, nor does it include any benefits not covered by Medicare, such as long-stay SNF claims or nursing home care. We used the 2015 MCBS to estimate the cost of these two components for the Medicare-eligible population. The MCBS collects data on healthcare services received by Medicare beneficiaries who are covered by Medicare or non-Medicare payers, such as the long-term care cost, for both fee-for-service (FFS) and non-FFS members, as well as for Medicare beneficiaries dually covered by other health plans, such as Medicaid.

Due to a lack of readily available data for the PD population younger than 65 who were either uninsured or covered by insurance types other than private insurance or Medicare, we imputed the cost of this relatively small population with the cost for the same age and gender strata from the Optum claims data for the privately insured. To account for the potential cost differences between the privately covered and the non-privately covered, we estimated a generalized linear model (GLM) with the gamma distribution and a log link to obtain the cost ratio between the private and the non-private groups and used the ratio to downward adjust the average cost per-privately insured by 28.6% for the other group.

PWPs in the claims data were identified based on at least one inpatient claim or at least two non-inpatient claims with PD; or with at least one outpatient claim and a prescription drug claim for an antiparkinsonian drug (this was only feasible in the private claims analysis). To account for the possibility of PWP being misdiagnosed, and to reflect the heterogeneous nature of PD, we included diagnosis codes that explicitly corresponded to PD diagnosis and other neurodegenerative conditions that could potentially be assigned to PWP prior to, or subsequent to receiving a PD diagnosis. These corresponding codes included Parkinson’s and Parkinsonism (ICD-9/ICD-10: 332.0/G20), secondary Parkinsonism (332.1/G21), dementia with Lewy bodies [DLB] (331.82/G31.83), striatonigral degeneration (333.0/G23.1), progressive supranuclear ophthalmoplegia [Steele-Richardson-Olszewski] (333.0/G23.2), degenerative disease of basal ganglia, unspecified (333.0/G90.3), and cortical basal degeneration [CBD] (331.6/G31.85).

To avoid excluding a potentially large number of false negative PD cases, we used this more inclusive list of diagnosis codes. As shown by Willis et al. using Medicare claims data, as many as 40% of identified PD patients never see a neurologist, let alone a Movement Disorder subspecialist^33^. It is generally accepted that non-neurologist physicians are insensitive in terms of detecting PD and poor at accurate classification of PD and related disorders. Accurate diagnosis and classification of PD and related disorders is often challenging, particularly in early disease phases, even by neurologists^35^. Autopsy series correlating clinical diagnosis of the more common PD mimics—progressive supranuclear palsy (PSP), multiple system atrophy (MSA), DLB—demonstrate that a substantial fraction of these individuals actually have PD^36–39^. As several of these studies are based on national brain bank collections and where it is likely that patients’ families were encouraged to donate by subspecialty Movement Disorders neurologists, these results likely represent floor estimates of PD classified as PD mimics. An approach similar to ours was used in the Horsfall et al. study of PD incidence trends in Great Britain, which used the large, primary care based Health Improvement Network database^40^. Our diagnostically inclusive approach may introduce a modest amount of additional uncertainty to our study sample than restricting diagnosis codes just to PD per se, but is more likely to give a realistic estimate of the PD cost.

### Indirect costs

Future earnings loss due to premature deaths attributable to PD was estimated using Centers for Disease Control and Prevention Wide-ranging Online Data for Epidemiologic Research (CDC WONDER) data and the Medicare Standard Analytical Files. To calculate loss in earnings, we first estimated the number of premature deaths associated with PD and then multiplied that number by an estimate of the present value of future earnings. We computed the net present value (NPV) of future earnings for men and women by age group to estimate the national productivity loss of early mortality associated with PD (Supplementary Table 3). The approach incorporates information on average annual earnings, takes into account labor force participation rates and mortality rates for men and women in the U.S., and assumes a productivity growth rate of 1% and a discount rate of 3%, a rate often used in public health studies^41–43^. We limited our calculation of earnings loss to adults 18–74 years of age (i.e., loss in earnings is assumed to be 0 for individuals who die prematurely due to PD at age 75 and above). It is important to highlight that all of the inputs in calculating earnings loss were based on publicly available statistics for the general U.S. population. We were not able to incorporate PD-specific information on earnings and employment due to a lack of available data.

For insights into the indirect and non-medical costs of PD, we designed and implemented a primary survey to estimate cost due to reduced labor market participation, productivity loss for those in the labor force and not in the labor force, cost of providing disability supplemental income such as Social Security Income (SSI) and Social Security Disability Insurance (SSDI), and the key items of non-medical costs of PD, such as the cost of hiring professional non-medical caregivers to assist with daily living, home modification costs, and increased transportation costs. A key purpose of the survey is to help understand the extent of family caregiver burden, which is a critical component of the indirect cost burden of PD. The survey instrument that included consent statement was reviewed by New England IRB and a copy of the survey instrument is available upon request.

The survey was administered electronically via two separate modes: one through The Michael J. Fox Foundation for Parkinson’s disease (MJFF) Fox Insight online survey platform, another to a broader audience of constituents of the Unified Parkinson’s Advocacy Council (UPAC) network, comprised of representatives from the state, regional, and national Parkinson’s organizations (more details of Fox Insight and UPAC can be found at https://www.michaeljfox.org/fox-insight and https://www.michaeljfox.org/news/unified-parkinsons-advocacy-council). Fox Insight is an online clinical study of people with PD and age-matched control volunteers providing the research community insight into the PD lived experience^44^. The survey account was established in the online survey vendor, Qualtrics.

Combining the Fox Insight survey sample and the UPAC sample, a total of 6,593 households responded to the survey. Among these, 4,722 (71.6%) completed the survey according to the electronic recording. Additional quality checks revealed that among 4,722 observations that were marked as “completed”, there were 105 respondents who did not have answers for a single question and 69 observations that answered that they do not have PD or don’t know anyone with PD. Eliminating these from the dataset reduced the sample size to 4,548 respondents. As the survey was based on a convenience sample, we calculated a survey weight for each PWP and reweighted the survey responses to represent the distribution of the entire U.S. PD population. Weights reflect the inverse probability of the survey counts relative to PD prevalence counts obtained from applying the PD prevalence rates by age and gender dimensions to the 2017 U.S. population age-gender counts. If the weight for a given age-gender multiplied by survey counts in the same age-gender strata, then it will yield the total number of people with PD in that strata. Prevalence rates were obtained from MEPS and MCBS data. See Supplementary Tables 4–8 for the characteristics of the survey sample.

### Future projections

We projected the future prevalence of PD by applying the estimated age- and gender-specific PD prevalence rate in 2017 to U.S. Census population projections for years 2018–2037. The projected PD prevalence based on this approach factored in population growth and demographic changes in population and assumed that current PD incidence and mortality rates remain constant during this period. Combining the projected future PD prevalence with the estimated 2017 per PWP burden by cost component, we also projected the future overall impact of PD through year 2037. We did not account for inflation, any potential changes in healthcare utilization or price changes due to changes in treatment intensity, etc. Neither did we apply a discount rate as these estimates represent the projected future burden in real 2017 dollars.

### Reporting summary

Further information on experimental design is available in the Nature Research Reporting Summary linked to this article.

## Supplementary information

Supplementary Tables

Reporting Summary

## Data Availability

Claims data and primary survey response data are either under DUA restrictions or are proprietary. Publicly available data sources include Medical Expenditure Panel Survey (MEPS) and U.S. Census population projections. MEPS is available at AHRQ: https://www.meps.ahrq.gov/ and U.S. Census population projection is available at: https://www.census.gov/programs-surveys/popproj.html.

## Code availability

Code used for analysis of publicly available data is available upon request from the authors.
