# Supplementary material for: Current and projected future economic burden of Parkinson’s disease in the U.S
Source: NPJ Parkinsons Dis. 2020 Jul 9;6:15. doi: 10.1038/s41531-020-0117-1 (PMC7347582; doi:10.1038/s41531-020-0117-1)
Supplement: Supplementary file 1 — Supplementary Tables [file 41531_2020_117_MOESM1_ESM.pdf]

Supplementary Information File for "Current and Projected Future Economic Burden of Parkinson's Disease in the U.S."

Supplementary Table 1. Parkinson's disease prevalence by population characteristics (in 2017)

	No. of Persons Estimated to Have PD	Population	Prevalence
**Age**			
≤49	17,000	212,270,000	0.01%
50-64	184,000	63,810,000	0.29%
65-74	385,000	28,860,000	1.33%
≥75	452,000	20,779,000	2.18%
**Gender**			
Male	595,000	160,355,000	0.37%
Female	443,000	165,364,000	0.27%
**Insurance**			
Private	77,000	176,965,000	0.04%
Medicare	919,000	56,213,000	1.63%
Other*	42,000	92,541,000	0.05%

Source: Author's calculations using 2011-2015 MEPS, 2015 MCBS, and Census population projection for 2017.
*Other includes Medicaid, other insurance, and uninsured.

Supplementary Table 2. Comparison of age, gender, & race/ethnicity between Parkinson's disease and comparison groups by data source

		PD		Comparison	
		N	Percent	N	Percent
**Optum Claims Data**					
Gender	Male	1,575	60.5%	15,750	60.5%
	Female	1,029	39.5%	10,290	39.5%
Age Group	≤49	408	15.7%	4,080	15.7%
	50-64	2,196	84.3%	21,960	84.3%
	65-74	N/A	N/A	N/A	N/A
	≥75	N/A	N/A	N/A	N/A
Race/Ethnicity	NH White	1,803	69.2%	18,030	69.2%
	NH Black	95	3.7%	950	3.7%
	Hispanic	176	6.8%	1,760	6.8%
	Other	246	9.4%	2,460	9.4%
	Unknown	284	10.9%	2,840	10.9%
**Medicare 5%**					
Gender	Male	12,102	54.0%	121,020	54.0%
	Female	10,308	46.0%	103,080	46.0%
Age Group	≤49	209	0.9%	2,090	0.9%
	50-64	1,654	7.4%	16,540	7.4%
	65-74	7,406	33.1%	74,060	33.1%
	≥75	13,141	58.6%	131,410	58.6%
Race/Ethnicity	NH White	19,579	87.4%	195,790	87.4%
	NH Black	1,311	5.9%	13,110	5.9%
	Hispanic	418	1.9%	4,180	1.9%
	Other	888	4.0%	8,880	4.0%
	Unknown	214	1.0%	2,140	1.0%
**Medicare Current Beneficiary Survey**					
Gender	Male	97	55.1%	970	55.1%
	Female	79	44.9%	790	44.9%
Age Group	≤49	6	3.4%	60	3.4%
	50-64	10	5.7%	100	5.7%
	65-74	45	25.6%	450	25.6%
	≥75	115	65.3%	1,150	65.3%
Race/Ethnicity	NH White	144	81.8%	1,140	81.8%
	NH Black	11	6.3%	110	6.3%
	Hispanic	19	10.8%	190	10.8%
	Other	2	1.1%	20	1.1%
	Unknown	N/A	N/A	N/A	N/A

Source: Author's calculations using the 2016 Optum claims, 2015 Medicare Standard Analytical File 5% sample claims, and 2015 Medicare Current Beneficiary Survey (MCBS).

Supplementary Table 3. Estimated net present value of the future earnings loss for premature deaths associated with Parkinson's disease

	Estimated Number of Premature Deaths	Estimated Present Value of Future Earnings/Death ($)	Estimated NPV (in Million $s)
**Males**			
≤49 years	18	909,826	16
50-64 years	2,139	345,062	738
65-74 years	13,511	57,337	775
**Females**			
≤49 years	171	769,680	132
50-64 years	2,219	283,802	630
65-74 years	5,335	40,774	218
**Overall**	**23,393**	**107,214**	**2,508**

Source: Lewin analyses of 2014-2016 CDC Wonder and 2015 Medicare 5% sample claims data. Death rates for ≥65 were derived from Medicare 5% data. Death rates for <65 non-PD population were derived from CDC WONDER data. Death rates for <65 PD population are estimated. Average earnings by age and gender obtained from Bureau of Labor Statistics.

Supplementary Table 4. Sample breakdown by respondents' self-description from the survey

Which of the following <u>best describes you</u> (the person who is responding to the survey)?	Freq.	Percent
A person with PD	3,098	67.1
A care partner for someone who has PD	1,264	27.4
A family member of someone who has PD, but not a care partner	180	3.9
A close friend of someone who has PD, but not a care partner	6	0.1
Sub-total	4,548	
Do not have PD and do not know anyone with PD	69	1.5
Total number of respondents	4,617	

Source: Primary data collected through the PD Impact Survey

Supplementary Table 5. Disease duration for persons with Parkinson's disease

	Unweighted		Weighted	
	Frequency	Percent	Frequency	Percent
N	4,548	100	1,037,211	100
Less than 1 year	206	4.5	40,474	3.9
1-5 years	1,537	33.8	329,114	31.7
5-10 years	1,256	27.6	289,176	27.9
10-15 years	641	14.1	153,504	14.8
15 or more	539	11.9	140,468	13.5
Missing	369	8.1	84,476	8.1

Source: Primary data collected through the PD Impact Survey

Supplementary Table 6. Percentage of persons with Parkinson's disease who spent time at a long-term care facility in the past 12 months

	Unweighted		Weighted	
	Frequency	Percent	Frequency	Percent
N	4,548	100	1,037,211	100
Private (alone or with someone)	4,284	94.2	955,084	92.1
Nursing home	192	4.2	65,983	6.4
Hospice facility	25	0.55	7,726	0.74
Active adult or senior living community	183	4.0	59,931	5.8
Other "long-term" care facility	115	2.5	35,815	3.5

Source: Primary data collected through the PD Impact Survey

Supplementary Table 7. Percentage of persons with Parkinson's disease who received unpaid care from a care partner in the past 12 months

		Unweighted		Weighted	
		Frequency	Percent	Frequency	Percent
Received Care from a Primary Care Partner (PCP)	Total	4,548	100	1,037,211	100
	Yes	2,677	58.9	648,185	62.5
	No	1,420	31.2	292,655	28.2
	Not applicable	451	9.9	96,371	9.3
Received Care from a Secondary Care Partner (SCP)	Total	4,548	100	1,037,211	100
	Yes	716	15.7	190,936	18.4
	No	2,748	60.4	594,623	57.3
	Not applicable	1,084	23.8	251,652	24.3

Source: Primary data collected through the PD Impact Survey

Supplementary Table 8. Baseline characteristics of the persons with Parkinson's disease and unpaid care partners

			Unweighted			Weighted		
			PWP	PCP	SCP	PWP	PCP	SCP
**All**			4,548	2,654	683	1,037,211	642,947	182,964
**Age**	≤49	N	162	204	373	16,695	37,276	84,142
		%	3.6	7.7	54.6	1.6	5.8	46.0
	50-64	N	1,335	811	209	183,767	165,122	72,295
		%	29.4	30.6	30.6	17.7	25.7	38.5
	65-74	N	1,937	1,126	72	384,944	255,939	20,733
		%	42.6	42.4	10.5	37.1	39.8	11.3
	≥75	N	1,101	513	29	451,806	184,609	5,794
		%	24.2	19.3	4.2	43.6	28.7	3.2
	Missing	N	13	.	.	0	.	.
		%	0.3	.	.	0	.	.
**Gender**	Female	N	1,732	1,882	439	442,580	449,729	119,099
		%	38.1	70.3	61.3	42.7	69.4	62.4
	Male	N	2,764	783	240	594,631	196,100	62,793
		%	60.8	29.3	33.5	57.3	30.2	32.9
	Prefer not to say	N	52	12	37	0	2,357	9,044
		%	1.1	0.4	5.2	.	0.4	4.7
**Race/ethnicity**	White Non-Hispanic	N	3,989	2,371	585	921,638	574,498	157,285
		%	87.7	88.6	81.7	88.9	88.6	82.4
	Black Non-Hispanic	N	34	29	11	6,295	5,727	2,559
		%	0.7	1.1	1.5	0.6	0.9	1.3
	Other Non-Hispanic	N	138	93	51	30,081	22,670	13,099
		%	3.0	3.5	7.1	2.9	3.5	6.9
	Hispanic	N	126	66	20	27,277	17,198	5,855
		%	2.8	2.5	2.8	2.6	2.7	3.1
	Prefer not to say	N	261	118	49	51,920	28, 092	12,138
		%	5.7	4.4	6.8	5.0	4.3	6.4

Source: Primary data collected through the PD Impact Survey. PWP: Person with Parkinson's disease; PCP – received care from a primary care partner; and SCP – received care from a secondary care partner.
