# Supplementary material for: Current and projected future economic burden of Parkinson’s disease in the U.S
Source: NPJ Parkinsons Dis. 2020 Jul 9;6:15. doi: 10.1038/s41531-020-0117-1 (PMC7347582; doi:10.1038/s41531-020-0117-1)
Supplement: Supplementary file 2 — Reporting Summary [file 41531_2020_117_MOESM2_ESM.pdf]

nature research

Corresponding author(s): *Double-blind peer review submissions: write DBPR and your manuscript number here instead of author names.*

Last updated by author(s): *YYYY-MM-DD*

Reporting Summary

Nature Research wishes to improve the reproducibility of the work that we publish. This form provides structure for consistency and transparency in reporting. For further information on Nature Research policies, see Authors & Referees and the Editorial Policy Checklist.

Statistics

For all statistical analyses, confirm that the following items are present in the figure legend, table legend, main text, or Methods section.

n/a	Confirmed	
☐	☒	The exact sample size (*n*) for each experimental group/condition, given as a discrete number and unit of measurement
☒	☐	A statement on whether measurements were taken from distinct samples or whether the same sample was measured repeatedly
☒	☐	The statistical test(s) used AND whether they are one- or two-sided<br>*Only common tests should be described solely by name; describe more complex techniques in the Methods section.*
☒	☐	A description of all covariates tested
☒	☐	A description of any assumptions or corrections, such as tests of normality and adjustment for multiple comparisons
☒	☐	A full description of the statistical parameters including central tendency (e.g. means) or other basic estimates (e.g. regression coefficient) AND variation (e.g. standard deviation) or associated estimates of uncertainty (e.g. confidence intervals)
☒	☐	For null hypothesis testing, the test statistic (e.g. *F*, *t*, *r*) with confidence intervals, effect sizes, degrees of freedom and *P* value noted<br>*Give P values as exact values whenever suitable.*
☒	☐	For Bayesian analysis, information on the choice of priors and Markov chain Monte Carlo settings
☒	☐	For hierarchical and complex designs, identification of the appropriate level for tests and full reporting of outcomes
☒	☐	Estimates of effect sizes (e.g. Cohen's *d*, Pearson's *r*), indicating how they were calculated

Our web collection on statistics for biologists contains articles on many of the points above.

Software and code

Policy information about availability of computer code

Data collection	A mix of publicly available data and proprietary data obtained via DUAs and a primary survey. No special software is used to collect data. The online survey is deployed via two vendors (see description in the article).
Data analysis	SAS is used to analyze direct costs; STATA is used to analyze indirect costs.

For manuscripts utilizing custom algorithms or software that are central to the research but not yet described in published literature, software must be made available to editors/reviewers. We strongly encourage code deposition in a community repository (e.g. GitHub). See the Nature Research guidelines for submitting code & software for further information.

Data

Policy information about availability of data

All manuscripts must include a data availability statement. This statement should provide the following information, where applicable:

- Accession codes, unique identifiers, or web links for publicly available datasets
- A list of figures that have associated raw data
- A description of any restrictions on data availability

Claims data can be obtained via DUAs with various vendors (e.g., CMS, Optum). Publicly available data (Medical expenditure panel survey) can be downloaded online. Survey data are proprietary and not publicly available.

Field-specific reporting

Please select the one below that is the best fit for your research. If you are not sure, read the appropriate sections before making your selection.

☐ Life sciences ☒ Behavioural & social sciences ☐ Ecological, evolutionary & environmental sciences

For a reference copy of the document with all sections, see nature.com/documents/nr-reporting-summary-flat.pdf

Behavioural & social sciences study design

All studies must disclose on these points even when the disclosure is negative.

Study description	Persons with Parkinson's disease have higher medical care needs, premature work cessation, often miss work, and require assistance from paid and unpaid care partners, leading to significant direct and indirect economic costs. This study provides a comprehensive analysis of the economic burdens of PD in the U.S. (2017) and projections for the next two decades.
Research sample	Claims data: PD identified based on the ICD-9 /ICD-10 codes. ICD-9/ICD-10: 332.0/G20, secondary Parkinsonism (332.1/G21), dementia with Lewy bodies [DLB] (331.82/G31.83), striatonigral degeneration (333.0/G23.1), progressive supranuclear ophthalmoplegia [Steele-Richardson-Olszewski] (333.0/G23.2), degenerative disease of basal ganglia, unspecified (333.0/G90.3), and cortical basal degeneration [CBD] (331.6/G31.85) Survey: convenience sample.
Sampling strategy	Claims data: all identified cases included subject to age constraints (i.e., commercial claims are limited to age <65). Survey: convenience sample.
Data collection	Multiple data sources were used to estimate the costs of PD, including public and private administrative claims data, Medicare Current Beneficiary Survey, Medical Expenditure Panel Survey, and a primary survey (n=4,548) designed for this study.
Timing	Commercial claims are for 2016; Medicare claims - for 2015, Medical Expenditure Panel Survey (MEPS) data for 2011-2015; Medicare Current Beneficiary Survey (MCBS) for 2015
Data exclusions	na
Non-participation	na
Randomization	na

Reporting for specific materials, systems and methods

We require information from authors about some types of materials, experimental systems and methods used in many studies. Here, indicate whether each material, system or method listed is relevant to your study. If you are not sure if a list item applies to your research, read the appropriate section before selecting a response.

Materials & experimental systems

n/a	Involved in the study	
☒	☐	Antibodies
☒	☐	Eukaryotic cell lines
☒	☐	Palaeontology
☒	☐	Animals and other organisms
☒	☐	Human research participants
☒	☐	Clinical data

Methods

n/a	Involved in the study	
☒	☐	ChIP-seq
☒	☐	Flow cytometry
☒	☐	MRI-based neuroimaging
